# Is methane emission genetically the same trait in young bulls and lactating dairy cows?

**DOI:** 10.3168/jdsc.2025-0792

**Published:** 2025-07-03

**Authors:** B. Heringstad, K.A. Bakke

**Affiliations:** 1 Department of Animal and Aquacultural Sciences, Faculty of Biosciences, Norwegian University of Life Sciences, 1432 Ås, Norway; 2 Geno Breeding and AI Association, 2317 Hamar, Norway

## Abstract

It is of interest to examine whether methane (CH_4_) emission is genetically the same trait in young bulls and lactating dairy cows. The aim was therefore to estimate the genetic correlation between CH_4_ emissions for Norwegian Red young bulls and lactating cows. Measures of CH_4_ from GreenFeed (GF) were available from Geno's test station for young bulls and from GF units installed across 14 commercial dairy herds. Data from the years 2020 to 2023 were included. After data editing, the dataset consisted of 771,989 GF visits from 1,370 cows, and from the test station we had 112,071 GF visits from 244 young bulls. Each young bull had, on average, 40 d with CH_4_ data, measured at 11 to 12 mo of age. Each visit to the GF provided an estimate of the animal's daily CH_4_ emission originating from rumen fermentation. The traits analyzed were grams of CH_4_ per animal per day, calculated as the daily average. The mean (SD) for cows and young bulls were 406 (108) and 222 (46) g of daily CH_4_, respectively. A bivariate linear animal repeatability model was used to estimate (co)variance components. The estimated heritability (SE) of CH_4_ emissions was 0.39 (0.04) for cows and 0.49 (0.15) for young bulls, and the estimated genetic correlation (SE) between the 2 traits was 0.63 (0.22). The large SE reflects that the genetic correlation was estimated based on information from relatively few animals, and results should therefore be interpreted cautiously. However, the results suggest that phenotyping future young artificial insemination bulls is valuable for the genetic evaluation of methane emissions in Norwegian Red, even if CH_4_ emission is not exactly the same trait genetically in young bulls and lactating cows.

## Highlights

• Genetic variation for methane emission in the Norwegian Red breed was explored.
• Breeding for lower methane emissions is feasible.
• Genetic correlation of 0.63 between methane emission in young bulls and lactating cows.

## Graphical Abstract

**Figure:**
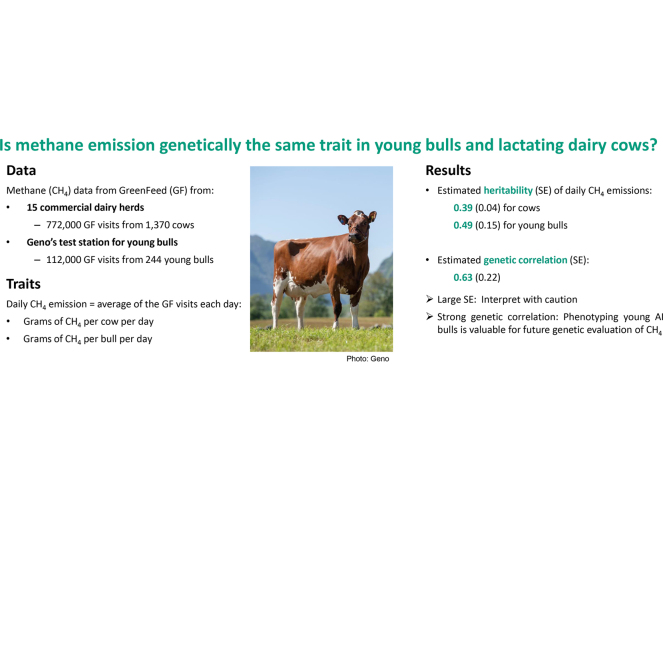
**Summary:** We estimated the genetic correlation to determine whether methane emission is the same trait in young bulls and cows in lactation. GreenFeed measures of methane from a test station for young bulls and from 14 commercial dairy herds were analyzed. The heritability of daily methane emission of 0.39 for cows and 0.49 for young bulls shows that breeding for lower methane is possible. The genetic correlation of 0.63 between the traits was far from 1, so the traits are not exactly the same. However, the substantial genetic correlation between the traits suggests that phenotyping young artificial insemination (AI) bulls is valuable for a future genetic evaluation of methane emissions in Norwegian Red cattle.

One way of reducing the environmental footprint of dairy production is by breeding. Selection for lower methane (CH_4_) emissions is possible (Lassen and Difford, 2020; Manzanilla-Pech et al., 2021). Most of the research on CH_4_ emissions in dairy cattle has been on lactating cows, but there are also a few genetic studies of CH_4_ emissions in young stock and bulls (Donoghue et al., 2016; Callegaro et al., 2022; Heringstad and Bakke, 2023).

Geno, the breeding organization for Norwegian Red, has established large-scale phenotyping of CH_4_ emissions in collaboration with 14 commercial dairy herds. GreenFeed (**GF**; C-Lock Inc., www.c-lockinc.com) units are in place in each of these herds, in addition to 1 GF unit installed at the test station for young bulls.

It is of interest to examine whether CH_4_ emission is genetically the same trait in young bulls and lactating dairy cows. The aim was therefore to estimate the genetic correlation between CH_4_ emissions for Norwegian Red young bulls and lactating cows.

Measures of CH_4_ from GF were available from 14 commercial dairy herds and from Geno's test station for young bulls. Data from the years 2020 to 2023 were included. The GF requires a minimum of 2 min with correct head position for good data, so only data from such visits were included. For the cows we included records from 5 to 350 DIM, from parities 1 to 9, where parities larger than 3 were grouped together. For further editing of the GF data for cows, we required a minimum of 5 records per GF unit per day, and a minimum of 10 records per cow. Measures outside the range from 100 to 800 g CH_4_ per day were excluded as outliers. For the GF data for young bulls, test-days with more than 9 records and animals with more than 9 records were included. Measures outside the range from 50 to 500 g CH_4_ per day were excluded as outliers.

After data editing, the dataset included 771,989 GF visits from 1,370 Norwegian Red cows. From the test station, we had 112,071 GF visits from 244 young bulls, measured at 11 to12 mo of age. The young bulls had an average of 40 d with CH_4_ data. Each visit to the GF provides an estimate of the animal's daily CH_4_ emission. The traits analyzed were grams of CH_4_ per animal per day, computed as the average of the individual visits each day, resulting in 222,133 records from cows and 10,514 records from young bulls.

The 2 traits were measured on 2 different groups of animals. However, a bivariate analysis is justified because the 2 groups are connected via pedigree. They are all from the same Norwegian Red breeding population. In the analyzed dataset, 90 Norwegian Red artificial insemination (**AI**) sires had offspring in both groups. Among the 244 young bulls with CH_4_ data, 151 had half-sisters with CH_4_ data, and 2 had daughters with CH_4_ data. Among the 1,370 cows with CH_4_ data, 598 (44%) had half-brother(s) with data, and 10 had a sire with data.

A bivariate linear animal repeatability model was used to estimate (co)variance components with the AI-REML procedure in the DMU software (Madsen and Jensen, 2013). The model for bulls had fixed effects of age and test-day, whereas the model for cows had fixed effects of parity, lactation week, and random herd-test-day effect. Both models had random effects of animal, permanent environment, and residual. The residual covariance was restricted to zero because the traits in question are measured on different animals. The joint pedigree file was traced back up to 5 generations and included a total of 14,081 animals. The distributions of CH_4_ measures from GF are in Figure 1. The mean (SD) of daily CH_4_ was 406 (108) g for cows and 222 (46) g for young bulls. The phenotypic levels were similar to previous reports (Wethal et al., 2022; Heringstad and Bakke, 2023).

**Figure 1:**
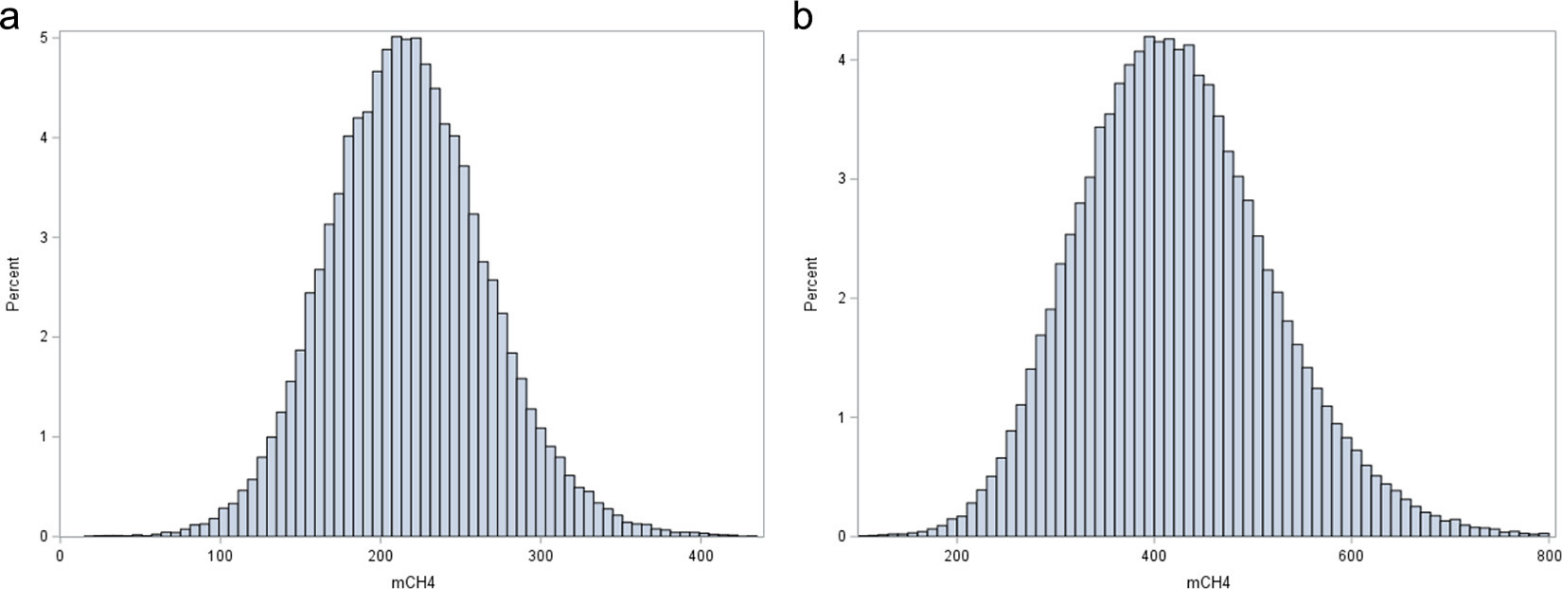
Distribution of phenotypic records of methane (CH_4_) emissions for Norwegian Red (a) young bulls and (b) lactating cows, measured as grams per day from GreenFeed. mCH4 = average of the GreenFeed visits each day.

Estimated variance components are given in Table 1. The corresponding heritability of CH_4_ emissions was 0.39 for cows and 0.49 for young bulls, with repeatability of 0.45 and 0.70, respectively. Heritability estimates were in agreement with previous estimates for Norwegian Red (Wethal et al., 2022; Heringstad and Bakke, 2023). Our estimates were in the upper range of reported heritability estimates in other dairy breeds. In a review, Lassen and Difford (2020) reported heritability estimates of methane for Holstein based on measures from sniffers ranging from 0.11 to 0.45, with most estimates between 0.21 and 0.27. Manzanilla-Pech et al. (2021) estimated genetic parameters for different methane traits using data from 4 countries, and the heritability was 0.21 for methane production (g/d), 0.30 for methane yield (g/kg DMI), and 0.38 methane intensity (g/kg ECM).

**Table 1:** Estimated permanent environment $\left(\sigma_{pe}^{2}\right),$ animal $\left(\sigma_{a}^{2}\right),$ herd-test-day $\left(\sigma_{htd}^{2}\right),$ and residual $\left(\sigma_{e}^{2}\right)$ variance with SE for daily methane (CH_4_) emissions for Norwegian Red young bulls and lactating cows, together with the corresponding heritability for CH_4._

Item	Young bulls		Cows	
	Estimate	SE	Estimate	SE
$\sigma_{pe}^{2}$	213	151	535	281
$\sigma_{a}^{2}$	510	172	3,958	430
$\sigma_{htd}^{2}$			1,894	29
$\sigma_{e}^{2}$	312	5	3,706	12
Heritability^1^	0.49	0.15	0.39	0.04

1 Heritability calculated as σa2σa2σa2σa2+σpe2+σhtd2+σe2.

The estimated genetic correlation (SE) between the 2 traits was 0.63 (0.22). As far as we know, this is the first study to estimate the genetic correlation between CH_4_ for dairy cows and young bulls. The large SE reflects that the genetic correlation was estimated based on information from relatively few animals, and the results should therefore be interpreted with caution. However, the results suggest that phenotyping future young AI bulls is valuable for the genetic evaluation of CH_4_ emissions in Norwegian Red, even if CH_4_ emission is not exactly the same trait genetically in young bulls and lactating cows.
